# Clinical accuracy of RIFLE and Acute Kidney Injury Network (AKIN) criteria for acute kidney injury in patients undergoing cardiac surgery

**DOI:** 10.1186/cc9960

**Published:** 2011-01-13

**Authors:** Lars Englberger, Rakesh M Suri, Zhuo Li, Edward T Casey, Richard C Daly, Joseph A Dearani, Hartzell V Schaff

**Affiliations:** 1 Division of Cardiovascular Surgery, Mayo Clinic, 200 First Street SW, Rochester, MN 55905, USA; 2 Division of Biostatistics, Mayo Clinic, 200 First Street SW, Rochester, MN 55905, USA; 3 Divison of Nephrology and Hypertension, Mayo Clinic, 200 First Street SW, Rochester, MN 55905, USA

## Abstract

**Introduction:**

The RIFLE (risk, injury, failure, loss of kidney function, and end-stage renal failure) classification for acute kidney injury (AKI) was recently modified by the Acute Kidney Injury Network (AKIN). The two definition systems differ in several aspects, and it is not clearly determined which has the better clinical accuracy.

**Methods:**

In a retrospective observational study we investigated 4,836 consecutive patients undergoing cardiac surgery with cardiopulmonary bypass from 2005 to 2007 at Mayo Clinic, Rochester, MN, USA. AKI was defined by RIFLE and AKIN criteria.

**Results:**

Significantly more patients were diagnosed as AKI by AKIN (26.3%) than by RIFLE (18.9%) criteria (*P *< 0.0001). Both definitions showed excellent association to outcome variables with worse outcome by increased severity of AKI (*P *< 0.001, all variables). Mortality was increased with an odds ratio (OR) of 4.5 (95% CI 3.6 to 5.6) for one class increase by RIFLE and an OR of 5.3 (95% CI 4.3 to 6.6) for one stage increase by AKIN. The multivariate model showed lower predictive ability of RIFLE for mortality. Patients classified as AKI in one but not in the other definition set were predominantly staged in the lowest AKI severity class (9.6% of patients in AKIN stage 1, 2.3% of patients in RIFLE class R). Potential misclassification of AKI is higher in AKIN, which is related to moving the 48-hour diagnostic window applied in AKIN criteria only. The greatest disagreement between both definition sets could be detected in patients with initial postoperative decrease of serum creatinine.

**Conclusions:**

Modification of RIFLE by staging of all patients with acute renal replacement therapy (RRT) in the failure class F may improve predictive value. AKIN applied in patients undergoing cardiac surgery without correction of serum creatinine for fluid balance may lead to over-diagnosis of AKI (poor positive predictive value). Balancing limitations of both definition sets of AKI, we suggest application of the RIFLE criteria in patients undergoing cardiac surgery.

## Introduction

The consensus criteria for acute kidney injury (AKI) developed by the Acute Dialysis Quality Initiative (ADQI) group, first published in 2004 [1] and named with the acronym RIFLE (risk, injury, failure, loss of kidney function, and end-stage renal failure), represent the first concerted effort to overcome the variety of different definitions for AKI. More recently, a modified version was proposed by the Acute Kidney injury Network (AKIN) [2]. The two definition systems for AKI differ in three major aspects. (I) In RIFLE the diagnosis is based on changes over a one-week period, while AKIN requires only changes within a designated 48-hour period. (II) Estimated glomerular filtration rate (eGFR) criteria are not included in AKIN. The percentage change from baseline serum creatinine (sCr) is identical in both definitions, aside from the additional criterion of an absolute sCr increment of ≥0.3 mg/dL within the AKIN stage 1 category. (III) Renal replacement therapy (RRT) in RIFLE was not assigned to a given severity class, whereas by definition all patients with RRT are placed in stage 3 by AKIN. Urinary output criteria are identical by both definition sets.

Our aim was to conduct a detailed comparison of RIFLE and AKIN criteria in a cardiac surgical patient cohort.

## Materials and methods

### Study population

After institutional review board approval (No. 09-001950, study specific informed consent was waived), we retrospectively investigated all patients who underwent cardiac surgical procedures with cardiopulmonary bypass (CPB) at Mayo Clinic Rochester within a three-year period (2005 to 2007). Patient data were recorded in a prospective computerized database. Patients with dialysis prior to surgery or with baseline sCr >3.0 mg/dL were excluded, as were those supported by extracorporeal membrane oxygenation (ECMO) preoperatively, patients undergoing cardiac/lung transplantation, assist device insertion or thoracoabdominal aortic repair. In accordance with Health Insurance Portability and Accountability Act (HIPAA) guidelines, patients who denied access to their medical records for the purpose of research were not considered for analysis. In patients who had more than one cardiac procedure with CPB during the study period at our institution (*n *= 42) only data related to the first operation were included. Patients who died intraoperatively or within 48 hours postoperatively were excluded (*n *= 30). In addition to available data each patient`s record was reviewed in order to document the following variables for study purposes: latest preoperative sCr values and sCr at each day postoperatively up to day seven (POD (postoperative day) 1 to 7). For baseline sCr the last value before surgery was recorded. If more than one sCr was measured per day in the postoperative period the highest recorded value was used for study purpose. One patient was excluded due to missing the preoperative sCr. The study cohort consisted of 4,839 patients. *Post-hoc *three patients were excluded who had RRT planned postoperatively.

### Data definitions

For the definition of AKI the sCr values at baseline and on POD 1 to 7 were used. AKI was defined by the RIFLE criteria using the maximal change in sCr and estimated glomerular filtration rate (eGFR) during the first seven postoperative days compared with baseline values before surgery. eGFR was calculated with the simplified Modification of Diet in Renal Disease (MDRD) formula [3]. Patients were stratified according to the highest RIFLE class R (Risk), I (Injury), or F (Failure) attained by sCr or eGFR criteria. Applying AKIN criteria, the strict definition was used for increments of sCr within a designated 48-hour interval (2). We compared daily sCr value with subsequent levels for the next two days until POD7 to create a moving 48-hour time window (preoperative baseline to POD 1 and 2, POD 1 versus POD 2 and 3, and so forth). The most severe degree of AKI was recorded as final AKIN stage. The RIFLE and AKIN definition criteria utilized are summarized in Table 1. The three thresholds for RIFLE and AKIN are called "classes" and "stages", respectively. All thresholds of RIFLE also included any patient who required RRT during the first seven days postoperatively (POD 1 to 7), whereas by definition these patients were located in stage 3 by AKIN. We did not use urine output criteria in defining AKI.

**Table 1:** Description of RIFLE [1] and AKIN [2] definition criteria for AKI used in analysis

Definition System	AKI		
**RIFLE**	**Class R (risk)**	**Class I (injury)**	**Class F (failure)**
Seven-day interval	sCr ≥1.5-fold increase or eGFR >25% decrease compared to baseline	sCr ≥2-fold increase or eGFR >50% decrease compared to baseline	sCr ≥3-fold increase or eGFR >75% decrease compared to baseline, or sCr increase to ≥4 mg/dL in setting of an increase of ≥0.5 mg/dL
**AKIN**	**Stage 1**	**Stage 2**	**Stage 3**
48-hour moving window	sCr ≥1.5-fold increase or ≥ 0.3 mg/dL	sCr ≥2-fold increase	sCr ≥3-fold increase compared to baseline, or sCr increase to ≥4 mg/dL in setting of an increase of ≥0.5 mg/dL, or RRT

AKI, acute kidney injury; eGFR, estimated glomerular filtration rate; RRT, renal replacement therapy; sCr, serum creatinine.

Definitions for outcome variables were the following: RRT as outcome variable included need for RRT during the entire postoperative hospital stay or within 30 days postoperatively. Operative mortality was in-hospital mortality or 30-day mortality. Prolonged intubation included those requiring ventilation for >24 hours postoperatively. Length of hospital stay was defined in days from index surgery to discharge.

### Statistics

Descriptive statistics for categorical variables are reported as frequency and percentage while continuous variables are reported as mean ± standard deviation or median (interquartile range) as appropriate. Categorical variables were compared between RIFLE classes or AKIN stages using Chi-square tests and continuous variables were compared using ANOVA or Kruskal-Wallis test where appropriate. Logistic regression models were used to predict the outcome variables mortality and prolonged intubation using RIFLE or AKIN classifications. Linear regression models were used to predict ICU length of stay and hospital length of stay using RIFLE or AKIN classifications. All tests were two-sided with the alpha level set at 0.05 for statistical significance. Data were analyzed using SAS 9.1 software (SAS Inc., Cary, NC, USA).

## Results

The final study cohort consisted of 4,836 consecutive patients, median age was 67 years (range 18 to 100 years), 34% (*n *= 1,633) were women. Table 2 presents detailed patient characteristics.

**Table 2:** Patient cohort characteristics

	Entire cohort
	(*n *= 4,836)
Demographics	
Age, years	64.4 ± 14.2
Female sex, n (%)	1,633 (34)
Medical history	
Diabetes, n (%)	981 (20)
Hypertension, n (%)	3,246 (67)
Chronic lung disease, n (%)	293 (13)
Extracardiac arteriopathy, n (%)	985 (20)
History of renal failure, n (%)	172 (4)
Baseline renal function	
Baseline serum creatinine, mg/dL	1.13 ± 0.29
Patients with baseline serum creatinine >2.0 mg/dL, n (%)	63 (1)
Baseline eGFR, mL/min/1.73 m^*2*^	68 ± 19
Baseline eGFR >60 mL/min/1.73 m^*2*^, n (%)	3,181 (66)
Baseline eGFR 31 to 60 mL/min/1.73 m^*2*^, n (%)	1,596 (33)
Baseline eGFR ≤30 mL/min/1.73 m^*2*^, n (%)	50 (1)
Preoperative cardiac status	
Prior cardiac surgery, n (%)	721 (15)
LVEF >60%, n (%)	2,405 (50)
LVEF 41-60%, n (%)	1,655 (34)
LVEF 21-40, n (%)	442 (9)
LVEF ≤20%, n (%)	55 (1)
LVEF (missing value), n (%)	279 (6)
History of Myocardial infarction, n (%)	1,027 (21)
Congestive heart failure, n (%)	775 (16)
NYHA functional class IV, n (%)	913 (19)
Cardiogenic shock, n (%)	34 (1)
Preoperative IABP, n (%)	77 (2)
Preop on inotropes, n (%)	114 (2)
Operative details	
CABG only, n (%)	1,258 (26)
CABG & Valve only, n (%)	566 (12)
Valve surgery, n (%)	1,196 (25)
Other/Combined surgery, n (%)	1,816 (37)
Elective surgery, (%)	3,947 (82)
Urgent surgery, n (%)	811 (17)
Emergent surgery & rescue, n (%)	78 (2)
CPB duration, minutes	85 ± 48
Cross-clamp time, minutes	58 ± 32
Patients with circulatory arrest, n (%)	220 (5)
Outcomes	
Intra/postop IABP, n (%)	173 (4)
Intra/Postop ECMO or VAD, n (%)	20 (0.4)
Revision for bleeding, n (%)	192 (4)
Operative mortality, n (%)	89 (1.8)
Hospital length of stay (alive), days	6 (5 to 8)

Data are mean ± SD and median (interquartile range) unless otherwise specified.

CABG, coronary artery bypass grafting; CPB, cardiopulmonary bypass; ECMO, extracorporeal membrane oxygenation; eGFR, estimated glomerular filtration rate; IABP, intra-aortic ballon pump; LVEF, left ventricular ejection fraction; NYHA, New York Heart Association; VAD, ventricular assist device.

In 65% of patients maximal sCr values during the first week postoperatively were detected during the first two postoperative days. In the entire cohort 96 (2.0%) patients had postoperative RRT, and 62/96 (65%) had RRT within the first seven days postoperatively.

Significantly more patients were diagnosed as AKI according to AKIN (*n *= 1,272, 26.3%) than by RIFLE (*n *= 915, 18.9%) criteria (*P *< 0.0001). Distribution of patients as well as agreement and disagreement within the different grades of AKI severity by RIFLE classes and AKIN stages are presented in Table 3.

**Table 3:** Agreement of RIFLE and AKIN definitions (numbers of patients and percentage of entire study cohort)

Definitions		*by AKIN definition*				
		no-AKI	stage 1	stage 2	stage 3	**total**
	
***by RIFLE definition***	no-AKI	**3,452**	466	0	3	3,921
		**(71.4%)**	(9.6%)		(0.06%)	(81.1%)
	class R	112	**582**	5	16	715
		(2.3%)	**(12.0%)**	(0.1%)	(0.33%)	(14.8%)
	class I	0	92	**50**	27	169
			(1.9%)	**(1.0%)**	(0.56%)	(3.5%)
	class F	0	1	2	**28**	31
			(0.02%)	(0.04%)	**(0.58%)**	(0.64%)
	**total**	3,564	1,141	57	74	**4,836**
		(73.7%)	(23.6%)	(1.2%)	(1.5%)	**(100%)**

Both definitions showed comparable and excellent univariate association to all outcome variables with worse outcome by increased severity of AKI, *P *< 0.001 for each variable (Table 4). Calculating the predictive ability of both definition systems, RIFLE class as well as AKIN stage were found to be significant predictors of increased mortality, prolonged intubation, prolonged ICU and hospital stay using multivariate analysis (*P *< 0.001 for all variables, Table 5). This was especially true for the mortality endpoint, where patients had an odds ratio of 4.5 (95% CI 3.6 to 5.6) for one class increase by RIFLE and an odds ratio of 5.3 (95% CI 4.3 to 6.6) for one stage increase by AKIN. Both definition sets of AKI showed good discrimination for the prediction of mortality as evaluated by the areas under the receiver operator characteristic curve (AUC): 0.80 (95% CI 0.75 to 0.85) for RIFLE and 0.82 (95% CI 0.77 to 0.87) for AKIN, respectively (Table 5).

**Table 4:** Outcomes by RIFLE and AKIN

RIFLE stage	No-AKI	class R	class I	class F	*P-value*
n (%)	3,921 (81.1)	715 (14.8)	169 (3.5)	31 (0.64)	
RRT, n (%)	8 (0.2)	33 (4.6)	37 (21.9)	18 (58.1)	<0.001
Mortality, n (%)	25 (0.64)	27 (3.8)	31 (18.3)	6 (19.4)	<0.001
Prolonged intubation (alive), n (%)	248 (6.4)	140 (20.3)	57 (41.3)	15 (60.0)	<0.001
ICU length of stay (alive), hours	25 (21 to 45)	46 (23 to 93)	105 (53 to 192)	188 (77 to 323)	<0.001
Hospital length of stay (alive), days	6 (5 to 7)	8 (6 to 11)	11 (8 to 21)	18 (10 to 27)	<0.001

**AKIN stage**	**No-AKI**	**stage 1**	**stage 2**	**stage 3**	***P-value***

n (%)	3,564 (73.7)	1,141 (23.6)	57 (1.2)	74 (1.5)	
RRT, n (%)	4 (0.1)	24 (2.1)	5 (8.8)	63 (85.1)	<0.001
Mortality, n (%)	19 (0.53)	30 (2.6)	7 (12.3)	33 (44.6)	<0.001
Prolonged intubation (alive), n (%)	211 (6.0)	204 (18.4)	17 (34.0)	28 (68.3)	<0.001
ICU length of stay (alive), hours	25 (22 to 44)	44 (22 to 90)	72 (29 to 147)	210 (120 to 356)	<0.001
Hospital length of stay (alive), days	6 (5 to 7)	7 (6 to 10)	10 (7 to 14)	19 (13 to 27)	<0.001

Data are presented as median (interquartile range) if not otherwise stated.

RRT, renal replacement therapy.

**Table 5:** Predictive ability of RIFLE and AKIN for outcome variables by logistic regression model

Outcome Variable	AKI Definition	Level	Odds Ratio (95% CI)	*P-value*	*AUC (95% CI)*
Mortality	RIFLE	per 1 class	4.5 (3.6 to 5.6)	<0.001	0.80 (0.75 to 0.85)
	AKIN	per 1 stage	5.3 (4.3 to 6.6)	<0.001	0.82 (0.77 to 0.87)
Prolonged intubation (alive)	RIFLE	per 1 class	3.3 (2.8 to 3.8)	<0.001	0.66 (0.64 to 0.69)
	AKIN	per 1 stage	3.3 (2.8 to 3.8)	<0.001	0.67 (0.64 to 0.69)

			**Estimate (95% CI)**	***P-value***	

ICU length of stay (alive), hours	RIFLE	per 1 class	61 (54 to 68)	<0.001	
	AKIN	per 1 stage	59 (53 to 66)	<0.001	
Hospital length of stay (alive), days	RIFLE	per 1 class	4.3 (3.9 to 4.8)	<0.001	
	AKIN	per 1 stage	4.1 (3.7 to 4.6)	<0.001	

For the sake of further comparison, an explorative *post-hoc *multivariate model was constructed including all categories of RIFLE and AKIN. Consistent results were seen for all outcome variables except mortality (*P *< 0.001 for AKIN and without statistical significance for RIFLE). In this explorative analysis, RIFLE seems to have a lower predictive value for mortality than AKIN.

Patients who required postoperative RRT (irrespective of staging by RIFLE or AKIN) had very poor outcomes with a mortality of 44.8% (Table 6). We found substantial disagreement between RIFLE and AKIN in classification of patients who required RRT (Table 4). Out of all patients with postoperative RRT, 63 (66%) had RRT within the first seven postoperative days and were consecutively classified in AKIN stage 3, whereas only 18 (19%) of these patients were categorized in the failure class by RIFLE. The other patients were distributed to other RIFLE classes (Table 4).

**Table 6:** Outcomes of patients who require postoperative renal replacement therapy

AKIN stage	No-RRT	RRT	*P-value*
*n *=	4,740	96	
Mortality, n (%)	46 (1.0)	43 (44.8)	<0.001
Prolonged intubation (alive), n (%)	419 (8.9)	41 (77.4)	<0.001
ICU length of stay (alive), hours	25 (22 to 48)	351 (163 to 517)	<0.001
Hospital length of stay (alive), days	6 (5 to 8)	26 (18 to 40)	<0.001

Data are presented as median (interquartile range) if not otherwise stated.

It is important to note that whereas 9.6% of patients classified as AKIN stage 1 had no-AKI by RIFLE, only 2.3% of patients in RIFLE class R had no-AKI by AKIN (Table 3). These groups were investigated in detail. Both populations demonstrated intermediate levels of the outcome variables (Table 7). Baseline characteristics of both of these groups showed significant differences compared to the patient groups staged as no-AKI. No patient in AKIN 1/RIFLE no-AKI group showed a sCr increase of ≥0.3 mg/dL within the seven postoperative days compared to preoperative baseline, whereas such an increment was observed in 84/112 (75%) patients in the RIFLE R/AKIN no-AKI group. AKI was over-diagnosed by the AKIN definition.

**Table 7:** Comparison of outcomes and baseline variables in patients detected as AKI by AKIN but not by RIFLE or not by AKIN but by RIFLE

	No-AKI	Group 1	Group 2	AKI	*P-value*	*P-value*
	**No-AKI by RIFLE AND AKIN**	**Patients with no-AKI by RIFLE AND AKIN stage 1**	**Patients with no-AKI by AKIN AND RIFLE class R**	**Patients in RIFLE class R AND in AKIN stage 1**	**Comparing group no-AKI vs group 1**	**Comparing group no-AKI vs group 2**

*n *=	3452	466	112	582		
Outcomes						
Mortality, (%)	18 (0.5)	6 (1.3)	1 (0.9)	15 (2.6)	0.05	0.6
Prolonged intubation (alive), n (%)	191 (5.6)	56 (12.2)	20 (18.0)	116 (20.5)	<0.001	<0.001
ICU length of stay (alive), hours	24 (21 to 44)	27 (22 to 60)	43 (23 to 91)	46 (23 to 94)	<0.001	0.004
Hospital length of stay (alive), days	6 (5 to 7)	7 (6 to 9)	7 (6 to 9)	8 (6 to 11)	<0.0001	0.006
Baseline variables						
Age, years	62.5 ± 14.4	67.9 ± 13.8	65.1 ± 12.8	-	<0.001	0.09
LVEF, %	60 ± 12	57 ± 13	55 ± 14	-	<0.001	<0.001
Baseline serum creatinine, mg/dL	1.09 ± 0.24	1.35 ± 0.37	0.86 ± 0.19	-	<0.001	<0.001
Prior cardiac surgery, n (%)	396 (11)	84 (18)	27 (24)	-	<0.001	<0.001

Data are presented as median (interquartile range) or mean ± SD if not otherwise stated.

Furthermore, separating patients according to initial change in sCr between baseline and POD 1, and focusing occurrence of AKI according to both definition sets, the largest disagreement between RIFLE and AKIN was found in the patient group who showed initial decrease of sCr (Table 8). In contrary, only marginal differences could be detected between final definition and staging in RIFLE or AKIN in the patients who had initial increase of sCr or stable sCr values (Table 8).

**Table 8:** Patients with acute kidney injury (AKI) according to serum creatinine changes between baseline and first postoperative day (POD)

		RIFLE				AKIN			
			
Creatinine change between baseline and POD 1	Entire cohort (*n *= 4,836)	No-AKI	class R	class I	class F	No-AKI	stage 1	stage 2	stage 3
Increase, n (%)	1,090 (22)	**523 (48)**	422 (39)	122 (11)	23 (2)	**515 (47)**	487 (45)	32 (3)	56 (5)
No change, n (%)	955 (20)	**792 (83)**	134 (14)	25 (2.6)	4 (0.4)	**775 (81)**	165 (17)	10 (1)	5 (0.5)
Decrease, n (%)	2,791 (58)	**2,606 (93)**	159 (6)	22 (0.8)	4 (0.2)	**2,274 (81)**	489 (18)	15 (0.5)	13 (0.5)
Total, n (%)	4,836 (100)	3,921	715	169	31	3,563	1,142	57	74

## Discussion

The widespread acceptance of consensus definitions for AKI is reflected in the increased utilization of both RIFLE and AKIN criteria in the literature. In order to progress further, establishment of a uniform definition for AKI applicable in a variety of patient populations is necessary. The aim of our study was to conduct an indepth comparison between both consensus definitions in a large retrospective cohort of patients undergoing cardiac surgery at a single center and to determine the influence of the three modifications made from RIFLE to AKIN. Our data demonstrate that important differences exist between the two classification schemes.

Existing comparative studies [4-10] are limited for different reasons. The main focus of comparison is most often the ability of both definition systems to predict outcome [4-10]. However, this was not the original intention of a consensus definition for AKI. The initial aim was to create a uniform definition to help researchers and ultimately clinicians to classify the extent of renal dysfunction and to improve prophylactic and therapeutic measures. Other limitations include various interpretations of the given criteria [4-10], heterogeneous time frames of observation [4-9], limitation of comparison to changes in sCr not including GFR thresholds [4-6,8-10], unknown RRT rates [4-6,10], and finally the lack of sufficient number of patients to determine relevant differences [7,9,10].

In our cardiac surgical cohort, significantly more patients were diagnosed as AKI by AKIN criteria than by the RIFLE definition set. This reflects one of the intentions of the Acute Kidney Injury Network to increase the sensitivity of AKIN compared to RIFLE [2].

Among patients defined as AKI by both definitions only limited disagreement occurred in the staging of severity grade. More interestingly and clinically important, the highest disagreement was found in the patient groups defined as AKI by RIFLE but not by AKIN and vice versa. In this situation either the definition system failed to classify the patients as having AKI or a patient was erroneously labeled with AKI but did not have the condition. It is important to analyze these patients further in detail.

The largest groups were identified in the lowest severity grades (Table 3). First, patients in both groups had poorer outcome endpoints (versus no-AKI patients), however, mortality rates did not differ significantly (Table 7). Second, baseline characteristics of both subgroups (vs. no-AKI) demonstrated that differences in outcome variables are possibly confounded by clinical factors other than AKI. Finally we determined that the overall differences between patients diagnosed as AKI by RIFLE or AKIN are mainly those who had an initial decrease of sCr from preoperative baseline to POD 1 (Table 8). In this group, post-operative sCr values that were lower than preoperative levels could serve as comparison in the 48-hour moving diagnostic window of AKIN. No patient in the AKIN 1/RIFLE no-AKI group had a sCr increase of ≥0.3 mg/dL above the pre-operative baseline within the entire observation period which is why the diagnosis of AKI is questionable (false-positive). The over-diagnosis of AKI by AKIN (accounting for almost 10% in our study cohort) is clearly caused by the moving 48-hour diagnostic interval and can be avoided only by correction of creatinine for fluid accumulation. This problem highlights the peculiarity of patients where positive fluid balance is present (that is, CPB with hemodilution). A physiological decrease in sCr following cardiac surgery is well understood [8], and our data demonstrate that this may have an important influence on predicting subsequent development of AKI (Table 8). Since no independent "gold standard" for the definition of AKI is available, we performed in our study the described three-step analysis.

The other patient group diagnosed as AKI class R by RIFLE but not by AKIN, frequently had an increase of sCr ≥0.3 mg/dL (84/122, 75%) compared to preoperative baseline. AKI could not be detected by AKIN due to the inability to obtain the critical threshold of ≥0.3 mg/dL within a 48-hour window [11]. Thus, the number of patients possibly misdiagnosed with AKI by AKIN is more than four-fold higher (9.6% vs. 2.3%) than by the application of the RIFLE criteria.

The moving 48-hour diagnostic window was introduced in AKIN [2] in order to overcome the limitation of RIFLE, that a diagnosis of AKI can be difficult when a baseline sCr is unavailable. The initially proposed solution used the revised MDRD formula with a suggested near lower limit of normal GFR (75 mL/minute/m2) to estimate baseline sCr [1]. This has subsequently been proven to perform well only when near-normal baseline kidney function is present [12]. It should be noted that preoperative sCr is available in most if not all patients undergoing cardiac surgery. Additional justification for the creation of a 48-hour diagnostic window was to detect an abrupt increase in sCr [2].

Logically, discriminating outcomes between patients with and without AKI may help to determine the validity of a definition/staging system. Several authors have discovered in a variety of patient cohorts that the thresholds of AKI severity defined either with RIFLE [13-19] or AKIN [20,21] were strongly associated with adverse patient outcome. We also confirmed these findings in our study. Interpretation of these findings is limited by focusing on renal function because a strong association does not prove a causal relationship. However, there is increasing evidence that the kidney is not simply a passive bystander in multiorgan dysfunction [22].

Patients who require postoperative RRT have a very poor outcome (Table 6). The different staging of patients who had RRT within the first seven days after surgery in the two definitions of AKI is very obvious (Table 4). Both classification schemes demonstrated good predictive value for outcome variables, however, the stepwise incremental mortality risk by AKI severity stage is better in AKIN. In this respect the predictive value of RIFLE may increase if all patients with RRT are staged in the highest possible class F, as done in the AKIN definition set. Notably, three patients in our study cohort staged in the highest severity class by AKIN but in RIFLE classified as no-AKI (Table 3) did require postoperative RRT.

The observation period for the diagnosis of AKI in our study was limited to the first seven days postoperatively. A longer time period might potentially alter our results, however we feel this is unlikely because a) the median postoperative stay of survivors was six days and b) AKI beyond POD 7 is more likely influenced by postoperative factors/complications than by renal injury during index surgery. This is in accordance with the ADQI VI consensus statement [23] where the authors advocate a separation into "early" (within the first seven days) and "late" cardiac surgery-associated AKI.

We have used sCr and GFR thresholds in calculating RIFLE classes. Besides limited accuracy of eGFR in AKI [24,25] it has been noted previously that the different thresholds given in RIFLE for sCr increase and eGFR decrease may lead to incongruent definition and staging [26]. We have shown very recently in our study cohort, that eGFR threshold (eGFR is directly dependent on sCr) in RIFLE is more sensitive to classify AKI patients than the sCr criteria [27]. This is in accordance with data recently extracted from a pediatric patient cohort [28]. In our study cohort, patients were classified as having AKI in 9.3% with the sCr criteria versus 18.9% with eGFR criteria, respectively. Thresholds for eGFR change in RIFLE have higher sensitivity to detect patients in class R and I, whereas changes in sCr show better sensitivity for RIFLE class F [27]. As proposed in the original RIFLE publication [1] our patients were allocated to the worst possible RIFLE class they attained by either one or another threshold. Using only the sCr criteria in RIFLE may alter our results considerably.

We did not use urinary output criteria in our retrospective study. These criteria are identical in RIFLE and AKIN for both amount of urinary output and reference time period [1,2]. By urinary output criteria both definition sets may diagnose and stage patients in corresponding severity classes which would not considerably influence our comparative study design (except for a few patients with RRT who may be located in a different RIFLE class by urinary output criteria). Nevertheless, the lack of urinary output data in our study has to be considered as a limitation, since there is a potential effect of the use or non-use of the urinary output criterion [11].

In our study the strict AKIN criteria were applied in the proscribed 48-hour moving window for diagnosis and staging of AKI. However, in the original AKIN paper the authors stated that "although diagnosis of AKI is based on changes over the course of 48 hours, staging occurs over a slightly longer time frame" [2]. Despite this difference in time frames, the likelihood is that it would not alter our results remarkably since the relevant difference between AKIN and RIFLE was not detected in the staging but in the diagnosis of AKI.

In a later publication [23], the ADQI group suggested for the use of the AKIN definition in clinical practice that the baseline reference sCr value in the postoperative period should be at least measured more than 24 hours after the start of surgery in order to prevent a diluted serum sample being used as reference. In our study, we did not apply strictly to this recommendation. However, first postoperative sCr values collected for study purpose were the sCr values measured the first day after surgery. The sCr values at ICU admission at the day of surgery were not considered in our study. We could demonstrate that the majority of patients (Table 8) undergoing cardiac surgery present lower sCr at the first postoperative day compared to preoperative baseline. A relevant proportion of patients may also have lower sCr compared to preoperative sCr on the following days. In this respect, the above mentioned 24-hour rule seems to be arbitrary. Nevertheless, it should be acknowledged when AKIN is used as definition criteria for AKI in cardiac surgical patients. In the cardiac surgical setting, when almost all patients have known preoperative sCr values it seems to be worthwhile to use this value as reference baseline throughout the first seven days postoperatively, which is in accordance with the RIFLE definition scheme.

One important finding of our study is the fact that fluid accumulation has to be addressed for accurate recognition and staging of AKI. In cardiac surgical patients the AKIN definition scheme may potentially lead to over-diagnosis of AKI. This is especially important for epidemiologic studies when sCr values at ICU admission after surgery serve as baseline values. It has been recently demonstrated, however, that dilution of sCr by fluid accumulation in critically-ill patients may in contrast also lead to underestimation of the severity of AKI and correction of sCr for fluid balance can improve recognition and staging [29].

Our findings are applicable for the cardiac surgical cohort and the detected differences between the both definition schemes of AKI may differ in other setting.

## Conclusions

In summary, balancing limitations and strengths of both consensus definitions of AKI (in the current versions), we favor the use of RIFLE criteria in patients undergoing cardiac surgery. Modification of RIFLE by staging all patients with acute RRT in the failure class F may improve the predictive value of this classification scheme. AKIN applied in patients undergoing cardiac surgery using the suggested 48-hour diagnostic window without correction of sCr for fluid balance may importantly lead to over-diagnosis of AKI. The quest for a uniform definition for AKI persists in its necessity and relevance [7,11,30].

## Key messages

• The AKIN definition criteria applied in patients undergoing cardiac surgery using the suggested 48-hour diagnostic window without correction of sCr for fluid balance may lead to over-diagnosis of AKI.

• Modification of RIFLE by staging all patients with acute RRT in the failure class F may improve the predictive value of this classification scheme.

• Balancing limitations and strengths of both consensus definitions of AKI (in the current versions), we favor the use of RIFLE criteria in patients undergoing cardiac surgery.

• Correction of sCr for fluid accumulation has to be addressed for accurate recognition and staging of AKI.

## Abbreviations

AKI: acute kidney injury; ADQI: Acute Dialysis Quality Initiative; AKIN: Acute Kidney Injury Network; AUC: areas under the receiver operator characteristic curve; CPB: cardiopulmonary bypass; ECMO: extracorporeal membrane oxygenation; GFR: glomerular filtration rate; HIPAA: Health Insurance Portability and Accountability Act; MDRD: Modification of Diet in Renal Disease; POD: postoperative day; RIFLE: risk, injury, failure, loss of kidney function, and end-stage renal failure; RRT: renal replacement therapy; sCr: serum creatinine.

## Competing interests

The authors declare that they have no competing interests.

## Authors' contributions

LE, RMS, ZL, and HVS were involved in the conception, design, acquisition of data, analysis and interpretation of data, drafting of the manuscript and revising it critically for important intellectual content and final approval of the version to be published. ETC, RCD, and JAD were involved in acquisition of data, interpretation of data, revising the manuscript critically for important intellectual content and final approval of the version to be published.

## References

[B1] Bellomo R Ronco C Kellum JA Mehta RL Palevsky P Acute Dialysis Quality Initiative workgroup Acute renal failure - definition, outcome measures, animal models, fluid therapy and information technology needs: the Second International Consensus Conference of the Acute Dialysis Quality Initiative (ADQI) Group Crit Care 2004 8 R204 R212 10.1186/cc2872 15312219 PMC522841

[B2] Mehta RL Kellum JA Shah SV Molitoris BA Ronco C Warnock DG Levin A Acute Kidney Injury Network Acute Kidney Injury Network: report of an initiative to improve outcomes in acute kidney injury Crit Care 2007 11 R31 10.1186/cc5713 17331245 PMC2206446

[B3] Levey AS Greene T Kusek JW Beck GL MDRD Study Group A simplified equation to predict glomerular filtration rate from serum creatinine [Abstract] J Am Soc Nephrol 2000 11 AO828

[B4] Lopes JA Fernandes P Jorge S Gonçalves S Alvarez A Costa e Silva Z França C Prata MM Acute kidney injury in intensive care unit patients: a comparison between the RIFLE and the Acute Kidney Injury Network classifications Crit Care 2008 12 R110 10.1186/cc6997 18755026 PMC2575599

[B5] Bagshaw SM George C Bellomo R ANZICS Database Management Committee A comparison of the RIFLE and AKIN criteria for acute kidney injury in critically ill patients Nephrol Dial Transplant 2008 23 1569 1574 10.1093/ndt/gfn009 18281319

[B6] Joannidis M Metnitz B Bauer P Schusterschitz N Moreno R Druml W Metnitz PG Acute kidney injury in critically ill patients classified by AKIN versus RIFLE using the SAPS 3 database Intensive Care Med 2009 35 1692 1702 10.1007/s00134-009-1530-4 19547955

[B7] Haase M Bellomo R Matalanis G Calzavacca P Dragun D Haase-Fielitz A A Comparison of the RIFLE and Acute Kidney Injury Network classifications for cardiac surgery-associated acute kidney injury: A prospective cohort study J Thorac Cardiovasc Surg 2009 138 1370 1376 10.1016/j.jtcvs.2009.07.007 19733864

[B8] Lassnigg A Schmid ER Hiesmayr M Falk C Druml W Bauer P Schmidlin D Impact of minimal increases in serum creatinine on outcome in patients after cardiothoracic surgery: do we have to revise current definitions of acute renal failure? Crit Care Med 2008 36 1129 1137 10.1097/CCM.0b013e318169181a 18379238

[B9] Yan X Jia S Meng X Dong P Jia M Wan J Hou X Acute kidney injury in adult postcardiotomy patients with extracorporeal membrane oxygenation: evaluation of the RIFLE classification and the Acute Kidney Injury Network criteria Eur J Cardiothorac Surg 2010 37 334 338 19692267 10.1016/j.ejcts.2009.07.004

[B10] Chang CH Lin CY Tian YC Jenq CC Chang MY Chen YC Fang JT Yang CW Acute kidney injury classification: comparison of AKIN and RIFLE criteria Shock 2010 33 247 252 10.1097/SHK.0b013e3181b2fe0c 19543147

[B11] Cruz DN Ricci Z Ronco C Clinical review: RIFLE and AKIN--time for reappraisal Crit Care 2009 13 211 10.1186/cc7759 19638179 PMC2717405

[B12] Bagshaw SM Uchino S Cruz D Bellomo R Morimatsu H Morgera S Schetz M Tan I Bouman C Macedo E Gibney N Tolwani A Oudemans-van Straaten HM Ronco C Kellum JA Beginning and Ending Supportive Therapy for the Kidney (BEST Kidney) Investigators A comparison of observed versus estimated baseline creatinine for determination of RIFLE class in patients with acute kidney injury Nephrol Dial Transplant 2009 24 2739 2744 10.1093/ndt/gfp159 19349297

[B13] Hoste EA Clermont G Kersten A Venkataraman R Angus DC De Bacquer D Kellum JA RIFLE criteria for acute kidney injury are associated with hospital mortality in critically ill patients: a cohort analysis Crit Care 2006 10 R73 10.1186/cc4915 16696865 PMC1550961

[B14] Uchino S Bellomo R Goldsmith D Bates S Ronco C An assessment of the RIFLE criteria for acute renal failure in hospitalized patients Crit Care Med 2006 34 1913 1917 10.1097/01.CCM.0000224227.70642.4F 16715038

[B15] Kuitunen A Vento A Suojaranta-Ylinen R Pettilä V Acute renal failure after cardiac surgery: evaluation of the RIFLE classification Ann Thorac Surg 2006 81 542 546 10.1016/j.athoracsur.2005.07.047 16427848

[B16] Ostermann M Chang RW Acute kidney injury in the intensive care unit according to RIFLE Crit Care Med 2007 35 1837 1843 10.1097/01.CCM.0000277041.13090.0A 17581483

[B17] Barrantes F Tian J Vazquez R Amoateng-Adjepong Y Manthous CA Acute kidney injury criteria predict outcomes of critically ill patients Crit Care Med 2008 36 1397 1403 10.1097/CCM.0b013e318168fbe0 18434915

[B18] Karkouti K Wijeysundera DN Yau TM Callum JL Cheng DC Crowther M Dupuis JY Fremes SE Kent B Laflamme C Lamy A Legare JF Mazer CD McCluskey SA Rubens FD Sawchuk C Beattie WS Acute kidney injury after cardiac surgery: focus on modifiable risk factors Circulation 2009 119 495 502 10.1161/CIRCULATIONAHA.108.786913 19153273

[B19] Bihorac A Yavas S Subbiah S Hobson CE Schold JD Gabrielli A Layon AJ Segal MS Long-term risk of mortality and acute kidney injury during hospitalization after major surgery Ann Surg 2009 249 851 858 10.1097/SLA.0b013e3181a40a0b 19387314

[B20] Ostermann M Chang R Riyadh ICU Program Users Group Correlation between the AKI classification and outcome Crit Care 2008 12 R144 10.1186/cc7123 19019254 PMC2646305

[B21] Thakar CV Christianson A Freyberg R Almenoff P Render ML Incidence and outcomes of acute kidney injury in intensive care units: a Veterans Administration study Crit Care Med 2009 37 2552 2558 10.1097/CCM.0b013e3181a5906f 19602973

[B22] Van Biesen W Lameire N Vanholder R Mehta R Relation between acute kidney injury and multiple-organ failure: the chicken and the egg question Crit Care Med 2007 35 316 317 10.1097/01.CCM.0000251987.16415.01 17197783

[B23] Hoste EA Cruz DN Davenport A Mehta RL Piccinni P Tetta C Viscovo G Ronco C The epidemiology of cardiac surgery-associated acute kidney injury Int J Artif Organs 2008 31 158 165 18311732 10.1177/039139880803100209

[B24] Van Biesen W Vanholder R Lameire N Defining acute renal failure: RIFLE and beyond Clin J Am Soc Nephrol 2006 1 1314 1319 10.2215/CJN.02070606 17699363

[B25] Delanaye P Cohen EP Formula-based estimates of the GFR: equations variable and uncertain Nephron Clin Pract 2008 110 c48 53 10.1159/000151436 18708727

[B26] Pickering JW Endre ZH GFR shot by RIFLE: errors in staging acute kidney injury Lancet 2009 373 1318 1319 10.1016/S0140-6736(09)60751-0 19376434

[B27] Englberger L Suri RM Schaff HV RIFLE is not RIFLE : on the comparability of results Crit Care 2009 13 429 10.1186/cc8175 20067601 PMC2811953

[B28] Zappitelli M Parikh CR Akcan-Arikan A Washburn KK Moffett BS Goldstein SL Ascertainment and epidemiology of acute kidney injury varies with definition interpretation Clin J Am Soc Nephrol 2008 3 948 954 10.2215/CJN.05431207 18417742 PMC2440279

[B29] Macedo E Bouchard J Soroko SH Chertow GM Himmelfarb J Ikizler TA Paganini EP Mehta RL for the Program to Improve Care in Acute Renal Disease (PICARD) study Fluid accumulation, recognition and staging of acute kidney injury in critically-ill patients Crit Care 2010 14 R82 10.1186/cc9004 20459609 PMC2911707

[B30] Mehta RL From acute renal failure to acute kidney injury: emerging concepts Crit Care Med 2008 36 1641 1642 10.1097/CCM.0b013e3181701481 18448916

